# Sex differences in the determination of prescribed load in ballistic bench press

**DOI:** 10.3389/fphys.2024.1293044

**Published:** 2024-01-12

**Authors:** Mohammad Izadi, Guglielmo Pillitteri, Ewan Thomas, Giuseppe Battaglia, Antonino Bianco, Marianna Bellafiore

**Affiliations:** Sport and Exercise Research Unit, Department of Psychology, Educational Sciences and Human Movement, University of Palermo, Palermo, Italy

**Keywords:** training load, propulsive phase, ballistic exercise, movement velocity, resistance training

## Abstract

**Introduction:** The objectives of the present study were twofold: first, to identify the specific relative load at which the concentric motion transforms into a purely propulsive action among women, and second, to compare the load-velocity relationships between men and women during the bench press throw.

**Methods:** Fourteen men and fourteen women participated in a test where they progressively increased the load until reaching their one-repetition maximum (1RM) in the bench press exercise. Linear regression models were employed to elucidate the relationships between load and velocity, as well as load and the propulsive phase (% of total concentric time). Additionally, ANCOVA was utilized to compare the linear regression models between men and women.

**Results:** The results revealed strong and linear associations between load and mean propulsive velocity (MPV) for both men and women, as well as between load and the propulsive phase. Notably, there were significant differences in MPV and the propulsive phase concerning load between men and women. Women transitioned into a fully propulsive concentric phase at approximately 80% of their 1RM, while men achieved this entirely propulsive phase at around 85% of their 1RM. Furthermore, women exhibited reduced velocities when handling lighter relative loads compared to men. Conversely, women demonstrated higher velocities when dealing with loads exceeding 85% of their 1RM in contrast to their male counterparts.

**Discussion:** These findings hold notable implications for prescribing bench press throw loads for women, which should differ from those recommended for men. Further studies are necessary to validate the efficacy of the proposed load recommendations.

## Introduction

Since athletes need to improve their performance through exercise, strength and conditioning coaches have focused on various techniques and training programs that can enhance athletic performance. One of the most common exercises is the bench press, which in turn helps athletes develop neuromuscular qualities and subsequently improve skills-related athletic performance (Sanchez-Medina et al., 2009). In this context, bench press throw is widely used to improve power-related capacities among athletes (Suchomel et al., 2018). Specifically, subjects need to accelerate the load throughout the movement from the beginning until the end of projection. Hence, since a variety of motions in athletic movements are required explosive motor actions, ballistic bench press exercise can assist athletes by maintaining velocity while improving strength, which in turn can enhance power-related capacities (Loturco et al., 2019).

Previous studies (Taber et al., 2016; Suchomel et al., 2018; Carvalho et al., 2022) demonstrated that prescribed training loads affect different aspects of muscle performance, in which very heavy weights (90%–95% 1RM) are employed to boost maximal strength (Suchomel et al., 2018), high loads (approximately 80% 1RM) are associated with muscle strength and hypertrophy improvements (Carvalho et al., 2022), and lighter loads (40%–60% 1RM) are used to enhance characteristics such as the rate of force development (RFD) and power-related qualities (Taber et al., 2016). Whereas ballistic bench press exercise can help improve athletic performance, there is still a concern related to prescribed loads. That is, an increase in load can lead to a decrease in velocity, such that if athletes tend to train in the presence of a high load (e.g., 80% 1RM), they cannot maintain propulsive acceleration throughout the concentric activation of exercise, which means movement velocity is too low. It is, therefore, impractical to project a loaded barbell into the air. In this vein, a recent study (Loturco et al., 2020) demonstrated that mean propulsive acceleration is close to zero during bench press throw after 80% 1RM, which suggests athletes cannot project a loaded barbell into the air. It was, therefore, proposed that strength and conditioning coaches prescribe any load below 80% 1RM if they tend to maintain the characteristics of ballistic exercise.

Given the substantial body of literature addressing sex differences in neuromuscular characteristics, including strength (Mata et al., 2016) and fatigability (Hicks et al., 2001; Davies et al., 2018), it is expected women demonstrate different responses compared to men in relation to ballistic bench press exercise. In particular, previous studies demonstrated that men with comparable training backgrounds exhibit a steeper slope in the load-velocity relationship compared to women (Torrejón et al., 2019; García-Ramos et al., 2021; Nieto-Acevedo et al., 2023). Thus, general equations that were formerly published to detect the propulsive phase of the concentric contraction might not be well-suited for women (Pareja-Blanco et al., 2020). Conducting research is essential to determining the appropriate load to maintain velocity in female athletes during a bench press throw. To the best of our knowledge, no study has been carried out to ascertain the specific relative loads at which women reach the point where the concentric action transforms into a purely propulsive phase during a bench press throw, meaning athletes are unable to project the load into the air. Therefore, the primary objective of the current study was to identify the specific relative load, represented as a percentage of 1RM, at which the concentric motion shifts into a purely propulsive action among women during the bench press. Additionally, to gain a deeper understanding of the differences between sexes in terms of the recommended load, we aimed to compare load-velocity relationships between men and women during the bench press throw. We hypothesized that female athletes would exhibit a different threshold load in the achievement of the pure propulsive phase during concentric action when compared to their male counterparts. It is also hypothesized that the change in velocity for a specific change in %1RM would be greater among men.

## Methods

### Subjects

While previous power analyses (Sreckovic et al., 2015) suggested that differences in mechanical variables (velocity, force, and power) could be detected with sample sizes as low as 3 to 9 participants, we conservatively enrolled 14 male collegiate athletes (age = 28.78 ± 3.19 years; body mass = 76 ± 6.23 kg; body height = 177.78 ± 4.99 cm) and 14 female collegiate athletes (age = 27.71 ± 2.33 years; body mass = 51.21 ± 8.91 kg; body height = 166.92 ± 3.33 cm) in the current study. The athletes possessed a range of 2–6 years of experience in weight training and were actively engaged in training, with 3 sessions per week, during the time of measurement for the study. These athletes had no history of musculoskeletal injuries in the past 6 months and any physical limitations that could affect the result of the study. Subjects were informed about the type of test and how to perform the bench press throw; however, were not informed regarding the outcomes of any their evaluations. Participants signed the informed consent before performing the test, and the present study was approved by the Institutional Review Board at the University of x.

### Procedures

Participants started to perform 1 RM test after a 10-min standardized warm-up, which consisted of jogging on a treadmill, stretching and upper-body joint mobilization exercises, and 1 set of 5 repetitions of bench press with a load of 8 kg (the weight of the Smith machine barbell). To perform the 1 RM test, the initial load was set at 8 kg for both male and female athletes. The external load was progressively increased by 10 and 5 kg for male and female athletes, respectively, until the achieved mean propulsive velocity (MPV) was less than 0.5 m s^-1^. Then, the load was increased by 5–1 kg to attain the precise estimation of 1RM bench press, such that 1RM was determined when an athlete could lift the heaviest load with the full extension of his/her elbow. Three, two, and one repetitions were executed for the lighter (MPV >1 m s^-1^), medium (0.65 m s^-1^ ≤ MPV ≤1 m s^-1^), and heaviest loads (MPV <0.65 m s^-1^), respectively. The rest period was 3 min for lighter and medium loads, while it was 5 min for the heaviest loads. The rest period between the repetitions executed with the same load was also 10 s (Sanchez-Medina et al., 2009; Torrejón et al., 2019).

Subjects performed the bench press throw in accordance with the method, which was extensively described in previous studies (Sanchez-Medina et al., 2009; Davies et al., 2018). The participants were first asked to execute the eccentric phase with control, holding a static position for at least one second at the end of this phase, ensuring that the bar lightly touched the chest. This was done to reduce the influence of the rebound effect and enhance measurement consistency. Following this, they were instructed to perform the concentric action with maximal effort. To provide safety for participants and give them feedback to keep their maximum velocity, two trained spotters were present on both sides of the barbell.

A Smith machine (JK Fitness Equipment) along with a dynamic measurement system (i.e., a linear velocity transducer that was sampled at a frequency of 1,000 Hz (T-Force System, Ergotech, Murcia, Spain)) was used to measure the MPV of the barbell during bench press throw. MPV was assessed throughout the concentric phase of the BP; in particular, the propulsive phase was determined as the portion of the concentric phase in which the acceleration of the movement was greater than the acceleration caused by gravity (i.e., g = 9.81 m s^-2^). The validity and reliability of the T-Force system were reported in previous studies (Sanchez-Medina et al., 2009; Perez-Castilla et al., 2019).

### Statistical analysis

Data were assessed for normality and homogeneity of variance using the Shapiro-Wilks test and Levene’s test, respectively. Linear regression models were employed to elucidate the association between load (%1RM) and MPV, as well as load (%1RM) and the propulsive phase (% of total concentric time). ANCOVA was utilized, with load as a covariate, to assess the sex-related differences in linear regression models. To better comprehend the distinctions between sexes in relation to dependent variables, we examined Cohen’s effect size (ES) along with its 95% confidence interval. This analysis was conducted across 5% increments, ranging from 20% 1RM to 100% 1RM. The criteria for interpreting the ES magnitude encompassed the following categories: trivial (2.0), small (0.2–0.6), moderate (0.6–1.2), large (1.2–2.0), and extremely large (>2.0) (Hopkins et al., 2009). Independent t-tests were also used to compare 1RM strength with respect to sex. Analyses were conducted using SPSS software version 26, and the level of significance was set at *p* < 05.

## Results

The 1RM strength was significantly different between men and women (*p* < 0.001; ES = 4.47; men = 88.71 ± 14.12 kg; women = 40.57 ± 5.63 kg).

**FIGURE 1:**
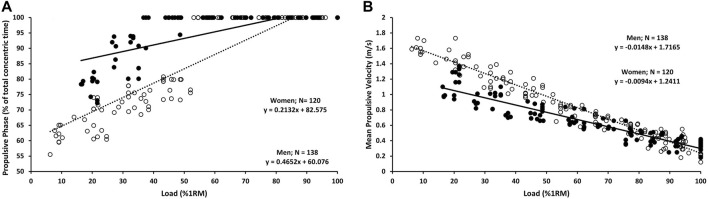
**(A)** Relationships between relative load (% 1RM) and Propulsive Phase for men (open dots and dashed line) and women (filled dots and solid line); **(B)** Relationships between relative load (% 1RM) and MPV for men (open dots and dashed line) and women (filled dots and solid line).

**FIGURE 2:**
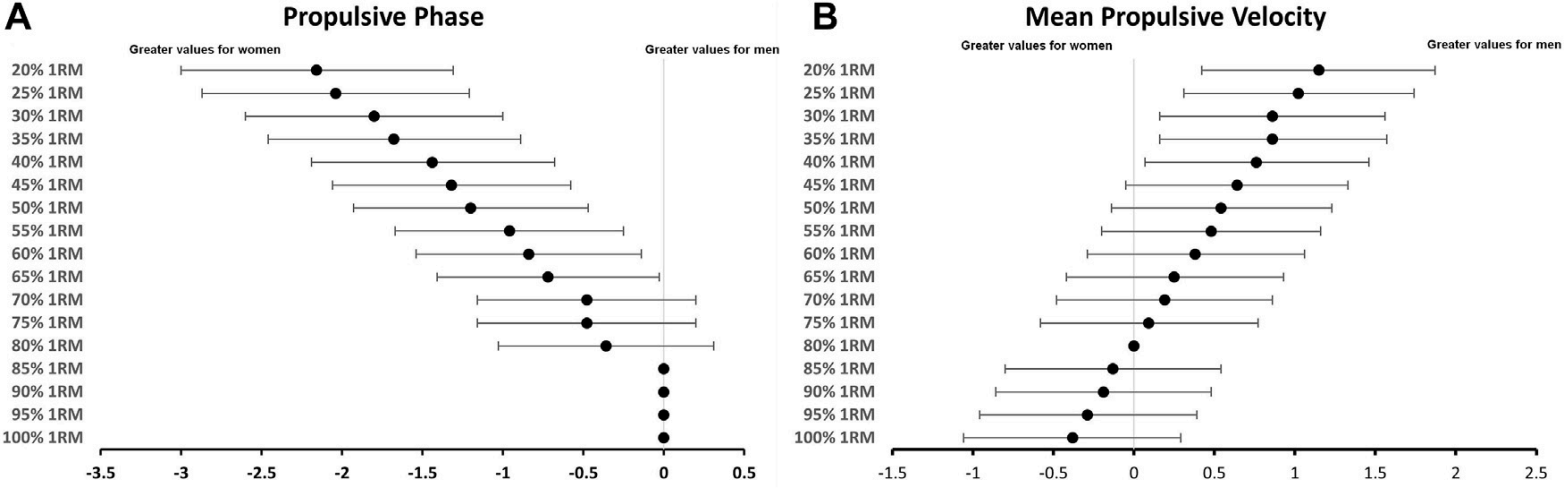
Effect size and its 95% confidence interval, represented as horizontal lines, are depicted in **(A)** for the Propulsive Phase and in **(B)** for the Mean Propulsive Velocity. Positive numbers indicate great values for men.

Table 1 displays the breakdown of concentric time into propulsive and braking phases at various percentages (from 20% to 100%) of 1RM for both men and women. The results from our linear regression models reveal a strong correlation between the load (1RM%) and the relative contribution of the propulsive phase to the total duration of the concentric lift for men (R^2^ = 0.817, *p* < 0.001) and women (R^2^ = 0.644, *p* < 0.001), as illustrated in Figure 1. A. The results of the ANCOVA analysis reveal significant differences between men and women concerning the relative contribution of the propulsive phase to the total duration of the concentric bench press throw (F = 43.431, *p* < 0.001). Specifically, at 20% 1RM, men accounted for 69% of the concentric time in the propulsive phase, whereas women demonstrated an 87% contribution to the propulsive phase at the same relative load. Notably, men reached a full propulsive phase at 85% 1RM, while women achieved the total propulsive phase at 80% 1RM, as detailed in Table 1. The effect size, along with its 95% confidence intervals, for the relative contribution of the propulsive phase to the total duration, is visually represented in Figure 2A.

**TABLE 1:** Sex differences in mean propulsive velocity (in meters per second) corresponding to various loads (as a percentage of 1RM) and the proportion of the propulsive phase’s contribution to the overall concentric duration.

Load (%1RM)	Men (*n* = 14)		Women (*n* = 14)	
	Propulsive phase (%)	MPV (m.s^-1^)	Propulsive phase (%)	MPV (m.s^-1^)
20	69	1.42	87	1.06
25	71	1.34	88	1.02
30	74	1.27	89	1
35	76	1.20	90	0.93
40	79	1.12	91	0.88
45	81	1.04	92	0.84
50	83	0.97	93	0.80
55	86	0.90	94	0.75
60	88	0.82	95	0.70
65	90	0.74	96	0.66
70	93	0.67	97	0.61
75	95	0.60	99	0.57
80	97	0.52	100	0.52
85	100	0.44	100	0.48
90	100	0.37	100	0.43
95	100	0.30	100	0.39
100	100	0.22	100	0.34

MPV, mean propulsive velocity.

The data analysis revealed a robust relationship between load (1RM%) and MPV in both men (R^2^ = 0.939, *p* < 0.001) and women (R^2^ = 0.855, *p* < 0.001), as depicted in Figure 1B. Furthermore, the results from the ANCOVA indicated significant differences between men and women regarding MPV (F = 19.745, *p* < 0.001). A comprehensive representation of MPV across various loads (ranging from 20% to 100% 1RM) can be found in Table 1. Additionally, Figure 2B visually illustrates the effect size of MPV, along with its corresponding 95% confidence intervals.

## Discussion

The aim of this study was to investigate the threshold loads at which women exhibit a solely propulsive phase during the bench press throw and to compare the load-velocity relationship between men and women. Our results revealed that, at approximately 80% of their 1RM, women transitioned into a completely propulsive concentric phase. In contrast, men exhibited this purely propulsive phase at around 85% of their 1RM. Furthermore, we observed a significant difference in the load-velocity relationship between men and women. Specifically, women displayed lower velocities when handling lighter relative loads compared to men. Conversely, women exhibited higher velocities when dealing with loads exceeding 85% of their 1RM, in comparison to their male counterparts.

In theory, ballistic exercises such as the bench press throw, which involve high-velocity movements, have the potential to enhance athletes’ power-related attributes, thereby elevating their performance in sports and competitive events (Loturco et al., 2020). However, it is important to note that the effectiveness of ballistic exercises in improving athletes’ performance can be influenced by the prescribed load, which alters their kinematics and kinetics. Specifically, heavier loads result in reduced velocity, ultimately negating the benefits of these exercises by preventing the effective projection of the load into the air. In this vein, prior studies (Sanchez-Medina et al., 2009; Moir et al., 2018; Loturco et al., 2020) have shown that when male athletes lift loads surpassing approximately 75%–80% of their 1RM, they are unable to harness the benefits of the ballistic bench press. This is because, at these specified loads, the concentric phase of the exercise primarily becomes propulsive, preventing athletes from effectively launching the loaded barbell into the air. In other words, the concentric contraction comprises both propulsive and braking phases. When lifting lighter loads (resulting in higher velocity), there is a prolonged braking phase during which acceleration exceeds that of gravity. However, when handling heavier loads (resulting in lower velocities), the braking phase diminishes, and acceleration falls below that of gravity (Sanchez-Medina et al., 2009). Consequently, athletes are unable to propel the barbell into the air. According to our data, women displayed a distinct propulsive phase at 80% of their 1RM, whereas men exhibited this phase at 85% of their 1RM. To delve deeper, when lifting at 20% 1RM, women demonstrated a significantly larger propulsive phase compared to men (87% for women and 69% for men). Interestingly, women exhibited an 87% propulsive phase even at just 20% of their 1RM, and from 35% to 75% of their 1RM, the propulsive phase ranged from 90% to 99%. This suggests that propelling the barbell into the air during this phase becomes particularly challenging. In line with prior research (Torrejón et al., 2019; García-Ramos et al., 2021; Nieto-Acevedo et al., 2023) advocating for the use of a specific equation to predict load-velocity relationships in women, our findings confirmed that women exhibited a higher value for the propulsive phase of concentric contraction than men.

In our current study, we observed a significant difference in the load-velocity relationship between men and women. This finding aligns with previous research (Torrejón et al., 2019; García-Ramos et al., 2021), which has consistently shown that women tend to exhibit lower velocities at lower relative loads compared to men. However, at higher relative loads (∼80% 1RM), women demonstrate higher velocities compared to men. While a limited number of studies have investigated load-velocity differences based on sex, these studies have utilized mean velocity and mean propulsive velocity as key variables in developing their models (Nieto-Acevedo et al., 2023). It is worth noting that while there is some variability among individuals, a clear pattern emerges when the movement becomes purely propulsive: both mean mechanical and mean propulsive variables converge, becoming indistinguishable. However, during phases that are not entirely propulsive, mean propulsive variables surpass mean mechanical variables in magnitude (Sanchez-Medina et al., 2009). Our data revealed that men demonstrated lower velocities compared to women when the bench press throw became entirely propulsive. The difference between the sexes may result from variations in muscle fiber types between men and women (Torrejón et al., 2019). Specifically, the higher prevalence of slow muscle fibers in women compared to men might contribute to their reduced speed when handling lighter relative loads (Mata et al., 2016). It can also stem from range of motion (ROM) (Nieto-Acevedo et al., 2023). In this vein, since men are taller and have longer limbs compared to women, prior studies (MOOKERJEE & RATAMESS, 1999; Martínez-Cava et al., 2019) have demonstrated that variations in ROM can affect RFD, activation, and synchronization of motor units. However, further research is needed to identify the mechanisms underlying these sex differences and to determine whether they are attributed to muscle fiber types and ROM.

The findings of the present study must be interpreted in light of certain limitations. Firstly, the athletes in our study were not engaged in supervised weight training at the time of data collection. Additionally, due to the inherent constraints associated with cross-sectional studies, it is imperative that the results of our study are validated through future research. Lastly, as our study exclusively involved athletes with prior resistance training experience, it is important to note that the findings may not apply to different athletic groups. Therefore, it is recommended to investigate the relative loads at which a concentric contraction shifts entirely to a propulsive phase among women participating in diverse sports disciplines.

### Practical applications

In the process of executing a proper barbell throw, athletes are required to maintain a persistent net positive force over an extended portion of the lift, thereby creating a more pronounced acceleration path throughout the upward phase of the motion. Furthermore, athletes need to decelerate the barbell to bring it to a complete stop during the concentric phase. The absence of a braking phase in this context renders it impossible to project the barbell into the air, as is typically the case in the traditional bench press (Loturco et al., 2020). As soon as the acceleration phase becomes entirely propulsive, it becomes unfeasible to project the barbell into the air. This point can be regarded as the 1RM for the bench press throw (Loturco et al., 2020). Therefore, coaches are advised to consider 80% of bench press-1RM for women and 85% of bench press-1RM for men as bench press throw-1RM when prescribing loads to athletes. In other words, 85% of bench press-1RM is equivalent to bench press throw-1RM. This approach aims to maintain the mechanical characteristics of ballistic exercises and optimize their performance.

## Conclusion

Our research revealed that women transitioned into a fully propulsive concentric phase at roughly 80% of their 1RM, while men achieved this entirely propulsive phase at approximately 85% of their 1RM. Additionally, a significant disparity emerged in the load-velocity relationship between men and women. To elaborate, women exhibited reduced velocities when handling lighter relative loads in contrast to men. Conversely, women demonstrated higher velocities when dealing with loads exceeding 85% of their 1RM, as compared to their male counterparts. These findings hold notable implications for prescribing bench press throw loads for women, which should differ from those recommended for men. Further studies are necessary to validate the efficacy of the proposed load recommendations.

## Data Availability

The raw data supporting the conclusion of this article will be made available by the authors, without undue reservation.
